# Winter wren populations show adaptation to local climate

**DOI:** 10.1098/rsos.160250

**Published:** 2016-06-29

**Authors:** Catriona A. Morrison, Robert A. Robinson, James W. Pearce-Higgins

**Affiliations:** 1 School of Biological Sciences, University of East Anglia, Norwich Research Park, Norwich NR4 7TJ, UK; 2 British Trust for Ornithology, The Nunnery, Thetford, Norfolk IP24 2PU, UK

**Keywords:** adaptation, climate change, temperature, winter wren, population change, micro-evolution

## Abstract

Most studies of evolutionary responses to climate change have focused on phenological responses to warming, and provide only weak evidence for evolutionary adaptation. This could be because phenological changes are more weakly linked to fitness than more direct mechanisms of climate change impacts, such as selective mortality during extreme weather events which have immediate fitness consequences for the individuals involved. Studies examining these other mechanisms may be more likely to show evidence for evolutionary adaptation. To test this, we quantify regional population responses of a small resident passerine (winter wren *Troglodytes troglodytes*) to a measure of winter severity (number of frost days). Annual population growth rate was consistently negatively correlated with this measure, but the point at which different populations achieved stability (*λ* = 1) varied across regions and was closely correlated with the historic average number of frost days, providing strong evidence for local adaptation. Despite this, regional variation in abundance remained negatively related to the regional mean number of winter frost days, potentially as a result of a time-lag in the rate of evolutionary response to climate change. As expected from Bergmann's rule, individual wrens were heavier in colder regions, suggesting that local adaptation may be mediated through body size. However, there was no evidence for selective mortality of small individuals in cold years, with annual variation in mean body size uncorrelated with the number of winter frost days, so the extent to which local adaptation occurs through changes in body size, or another mechanism remains uncertain.

## 1. Background

Species' distributions and range boundaries are widely regarded to be closely related to climate [1–3], indicative of limits in the evolutionary potential of species to adapt to climatic variation. Accordingly, species' range margins have generally shifted poleward or upward as climates have warmed [4–6]. Within their ranges, however, species vary in their phenotypic expression of traits across a gradient of environmental conditions as a result of local adaptation and/or plasticity [7–9]. Projected impacts of climate change upon species' extinction risk [10–12] have stimulated much interest in the potential for populations to exhibit evolutionary responses to climate change [13]. To date, this has particularly focused on phenological responses to changing environmental conditions, such as timing of breeding and migration, which are relatively easily measured traits with some fitness consequences [14]. In this case, the majority of studies suggest that much of the phenotypic responses observed results from individual plasticity [15–18], with relatively few studies providing evidence for local adaptation (but see [19,20]). Given that recent studies imply that the fitness consequences of mistimed breeding may be less severe than previously thought, at least in birds [21,22], there is a need to consider the evidence for populations exhibiting local adaptation to climate with respect to other mechanisms and traits.

Some of the closest links between climate and fitness are likely to operate through the frequency or magnitude of severe weather events that cause widespread mortality. For example, morphological changes that are consistent with directional selection occurring have been recorded in a number of populations in response to extreme events [23–27], although the evidence for consistent selection remains unclear [28]. The population abundance of many small resident passerines in temperate and boreal regions is influenced by winter weather severity, with cold weather associated with increased mortality and population decline [29–31]. As many of these species are relatively short-lived, with rapid generation times, we might therefore expect population responses to winter weather severity to be a good model with which to test evidence for local adaptation to climatic conditions. Given that body size is also related to tolerance to low temperatures, with smaller bodied individuals more susceptible for physiological reasons [23,25], we might also expect that any such adaptation is mediated through variation in individual body size.

Here, we use large-scale citizen-science population monitoring data of winter wren *Troglodytes troglodytes* populations along a strong climatic gradient in the UK to test whether spatial variation in population responses to winter weather severity shows evidence for local adaptation, and whether any such adaptation may be related to body size. The wren is a good model species in which to explore these relationships, as, being resident, annual wren survival is closely associated with winter cold [32,30], the main driver of annual variation in population growth [29]. Further, given low natal dispersal rates [33], which are likely to limit the exchange of individuals between populations over large spatial scales and short lifespans (approx. 2 years [30]), they are a species with high potential for relatively rapid evolutionary adaptation to occur.

## 2. Material and methods

### 2.1. Data collection

#### 2.1.1. Abundance data

This study used data from the BTO/JNCC/RSPB Breeding Bird Survey (BBS), which has been operating since 1994 with a mean of more than 2600 1 km^2^ squares surveyed per annum, selected according to a stratified random sampling protocol [34,35]. Each square is surveyed twice, once in the early breeding season (April to mid-May) and again in the late breeding season (mid-May through June) with all encounters recorded along two 1 km line-transects through the square. We used the maximum of the two counts within each 1 km^2^ as our measure of wren abundance for each year. To ensure that the models describe temporal, and not spatial, variation in population growth, BBS squares where wrens were recorded in fewer than 3 years (and therefore that cover only one temporal change) were removed from the dataset. As counts show considerable stochasticity in each square, we analysed data across 10 regions for which contemporary climate data were also available (electronic supplementary material, figure S1) and which provided sufficient samples for robust estimates of annual abundance and biometric data.

#### 2.1.2. Climate data

Previous analysis has demonstrated a strong link between annual wren survival and the number of winter frost days [30]. Therefore, we created a winter frost day (FD) index (1994–2011) for each of the 10 regions (electronic supplementary material, figure S1) by taking an average of the number of frost days per month across the autumn and winter (October to March) from http://www.metoffice.gov.uk/climate/uk/summaries/datasets. We calculated the regional mean number of winter frost days (FD_C_), as a measure of current climate, by taking the mean of the FD indices (1994–2011) for each region, and the historical regional climate (FD_H_), as the regional mean of the FD indices for the World Meteorological Office's international standard reference period (1961–1990).

#### 2.1.3. Biometric data

Wren biometric data were derived from individuals trapped by ringers as part of a national ringing (banding) programme [36]. Body mass (to the nearest 0.1 g) is routinely obtained by weighing individuals using spring or electronic balances, while wing length is measured using the maximum chord method from the carpus to the tip of the longest primary feather (millimetres). An individual's body mass varies dynamically in response to continually changing temperatures and other factors [37]. As we are interested in long-term responses across the population rather than direct plastic phenotypic responses to variation in winter weather conditions, we calculated annual mean weight and wing length from adult individuals caught only during the breeding season (April to June) in each region.

### 2.2. Statistical analysis

#### 2.2.1. Wren abundance and winter frost days

In order to examine the regional variation in annual population growth rate (*λ*) from 1994 to 2011 in relation to FD, a Poisson generalized linear mixed model (GLMM) was fitted using the glmer function in the lme4 package [38] in R v. 3.1 [39]. Annual counts were modelled as a function of region, FD and the interaction between these terms. Site (BBS square) and year were included as random effects in order to account for differences in coverage between years and the model was offset by the log of the count in the previous year to model population growth, rather than abundance. The count from the previous year was also included in the model to account for potential density-dependence in population growth rate [40]. To test the explanatory power of each of the fixed effects parameters in the model and their interaction, we used Akaike's information criterion (AIC); models with lower AIC were taken to provide a better explanation of the variation in the data. Thus, a large increase in AIC on variable removal (ΔAIC > 2, following Burnham & Anderson [41]) represents a notable deterioration in the model, providing ‘support’ for the importance of the variable that should be retained in the model. The output from this model was used to estimate a value for the number of winter frost days when population growth rate in each region (*λ*) was equal to 1 (i.e. population stability), as a single measure of population sensitivity to winter severity.

To test for evidence of local adaptation, this value was then correlated with FD_H_ as a measure of historic regional climate experienced by the population prior to the study, using a Pearson correlation. To confirm that our results are not an artefact of using this two-step approach, we repeated our analysis modelling the regional variation in annual population growth rate (*λ*) as a function of FD_H_, FD and the interaction between these terms. Site, year and region were included as random effects in order to account for differences in coverage between years, previous year's count was included as a fixed effect, and the model was offset by the log of the count in the previous year to model population growth, rather than abundance. Although this second model cannot be readily used to describe the close link between the measure of population sensitivity to winter severity (the number of frost days at which *λ* = 1) and historic regional climate (FD_H_), which is a function of both region-specific intercept and slope values, the output from this alternative model was analogous to the main results (electronic supplementary material, table S1).

In addition to modelling the effect of frost on population growth, we examined whether regional variation in mean abundance was also related to FD, again using a Poisson GLMM with a log link. Counts were modelled as a function of region, with site and year included as random effects in order to simply generate regional abundance estimates. These means were then correlated with the FD_C_ using Pearson correlation, using the same two-step approach described above.

#### 2.2.2. Wren biometrics and winter frost days

Finally, we examined the relationship between wren morphology and climate in an attempt to find a mechanistic link between population sensitivity to the number of winter frost days and regional climate. We first correlated regional variation in body mass and wing length of individuals caught in the breeding season (April to June) from 1994 to 2011 with FD_c_ to test whether spatial variation in body size is consistent with adaptation to the local climate. We then examined whether annual variation in body mass and wing length was driven by variation in FD using a general linear model (GLM) with normal errors and a log link function, fitted using the glm function in R [39]. The mean annual mass in each region and the equivalent wing length metric were modelled as a function of region, FD and the interaction between these terms. The model was offset by the log of the mass or wing length in the previous year to model proportional change, and annual mean regional abundance was also included as a fixed effect to control for possible density effects on body size. We focus our analyses on body mass as those involving wing length yielded similar results (see the electronic supplementary material).

## 3. Results

From 1994 to 2011, both the mean number of frost days across the UK and the national wren population index were highly variable. Wren populations declined in the last years of the study, during three cold winters (electronic supplementary material, figure S2), prior to which the population had increased, coincident with a period of mild winters. There was no significant trend in either the mean number of frost days across the UK (slope = 0.07 (±0.08 s.e.), *p* = 0.44) or the national wren population index (slope = 0.24 (0.76), *p* = 0.70), during this time period.

**Figure 1.**
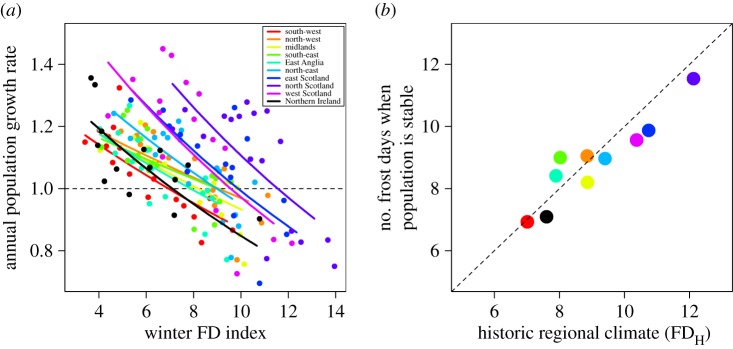
(*a*) Relationship between annual population growth rate and the winter FD index (average number of frost days per month (October to March) from 1994 to 2011). Each data point corresponds to an annual regional mean with annual population growth rate predicted from the results of a GLMM (table 1). Lines correspond with the predictions from the GLMM (table 1). (*b*) Relationship between the number of frost days when populations are stable (i.e. *λ* = 1) and the historic regional climate (FD_H_) (slope = 0.78 (±0.11 s.e.)). The number of winter frost days when the population is stable is predicted from the results of the GLMM (table 1) as plotted in (*a*), while the historic regional climate (FD_H_) is an overall mean of the winter FD index from 1961 to 2011.

As expected, there was a strong negative relationship between annual population growth rate (1994–2011) and FD across all 10 regions (mean slope = −0.045 (±0.003 s.e.); figure 1*a* and table 1). Critically, the number of winter frost days at which *λ* = 1 varied between regions (range = 6.9 (south-west) to 11.5 (east Scotland); figure 1), due to variation in the region-specific intercepts and the region × winter FD interaction. This FD value at stability was strongly correlated with FD_H_ (*r* = 0.93, *n* = 10, *p* < 0.001; figure 1*b*), with a close to 1 : 1 relationship; wren populations were stable if they were exposed to a winter of equal severity to the long-term average. Consequently, the effect of the winter FD index upon population growth varied significantly with FD_H_ (electronic supplementary material, table S1).

**Table 1.** Results of a GLMM of association between wren population growth rate and the winter FD index (average number of frost days per month (October to March) in 1994–2011) in each climate region (figure 1*a*). Count in the previous year was included in the model to account for potential density-dependence in population growth rate.

parameter	*χ*^2^	d.f.	*p*-value
region	114.2	9	<0.001
FD	50.8	1	<0.001
count in previous year	5151.0	1	<0.001
region × FD	85.5	9	<0.001

**Figure 2.**
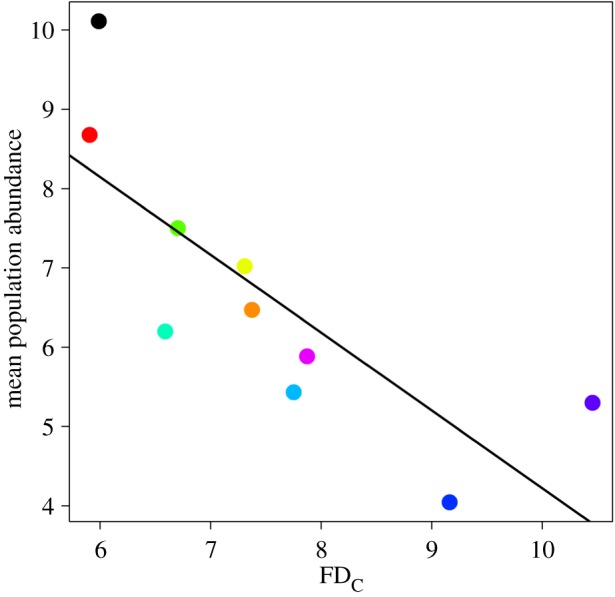
Relationship between population abundance and the regional mean number of winter frost days (FD_C_; slope = −0.98 (±0.27 s.e.)). Each data point corresponds to a regional mean, with FD_C_ calculated as the overall mean of the winter FD indices from 1994 to 2011, while the mean population abundance from 1994 to 2011 (number of wrens per BBS site) was predicted from the results of a GLMM (see Material and methods). Colours follow figure 1.

Despite this strong evidence for local adaptation, wrens were less common in colder regions of the UK (*r* = −0.78, *n* = 10, *p* = 0.009; figure 2). These effects were not a consequence of spatial variation in habitat composition between regions, as the same pattern was observed if data were included only from woodland sites, which represent wren core habitat [42] (electronic supplementary material, figure S3).

**Figure 3.**
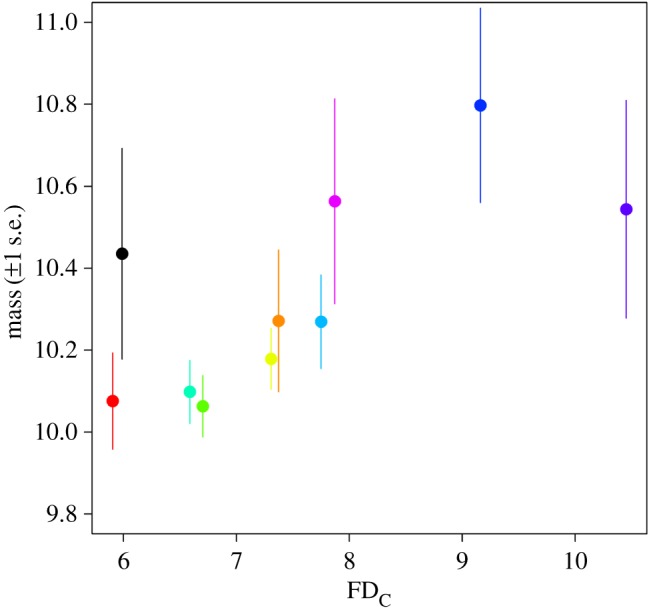
Relationship between mass and the regional mean number of winter frost days (FD_C_; slope = 0.12 (±0.04 s.e.)). Each data point corresponds to a regional mean, with mass calculated as a mean of the annual means and the number of winter FD (FD_C_) calculated as the overall mean of the winter FD indices from 1994 to 2011. Colours follow figure 1.

As expected, wren body mass was negatively correlated with FD_c_; wrens became heavier (*r* = 0.69, *n* = 10, *p* = 0.026; figure 3; electronic supplementary material, table S2) and had longer wings (electronic supplementary material, figure S4 and table S2) as FD_c_ increased. However, we found no evidence of a relationship between the annual relative change in mass (table 2) or wing length (electronic supplementary material, table S3) and FD; mean mass did not increase following frosty winters as would be expected if there were differential mortality of small individuals. There was also no evidence that the relationship between body size change and FD varied regionally (*p* = 0.76, table 2).

**Table 2.** Results of a GLMM of association between annual change in the mean body mass and the winter FD index (average number of frost days per month (October to March) in 1994–2011) in each climate region. Abundance was included in the model to control for possible population density effects on body size.

parameter	*χ*^2^	d.f.	*p*-value
region	0.99	8	0.99
abundance	0.33	1	0.57
FD	0.19	1	0.66
region × FD	4.94	8	0.76

## 4. Discussion

We identified the expected strong negative effect of winter severity (average number of winter frost days) upon wren populations, which was consistent across regions and matches previous studies [29,43]. The most likely environmental driver for this is reduced survival in cold winters [30,32]. Although the form of this relationship was broadly consistent between regions, there were significant differences which meant that the number of winter frost days at which individual populations achieved stability varied, with northern populations resilient to winters with up to 70% more frost days than southern ones. Crucially, there was a close correlation between historic regional climate (FD_H_) and the level of frost days at which each population is stable, indicating that each wren population was closely adapted to its local climate, as measured by the 1961–1990 mean.

Ours is one of only a small number of studies whose results are consistent with local adaptation occurring in population responses to climate (e.g. [18,19,44]). Previous studies of adaption in response to climate change, which have focused on the relationship between temperature and phenology [16–19,45], have found little evidence for evolutionary adaptation driving population-level responses to warming [16,17]. This could be due to methodological difficulties in identifying such local adaptation [46], or because phenological expression has a relatively weak link to individual fitness and population-level responses [21,22]. It seems likely that we have identified an adaptive response in wrens to local climate because we have been examining variation in an environmental trait that is closely linked to survival, and therefore to selection potential. Where phenological change has been shown to have a close link to fitness, as in the winter moth *Operophtera brumata*, then evolutionary responses have also been identified [20].

As expected and in accordance with Bergmann's rule, we found that wren body mass was approximately 5% lower in the warmest (south-west) than in the coldest (east Scotland) region. Large individuals are likely to be favoured in colder regions due to the thermal advantage of larger size and their ability to store more fat [25,47,48]. Despite this spatial link between climate and body size, we found no evidence for significant changes in body size to occur in relation to the number of winter frost days, suggesting that differential mortality of small individuals did not occur in cold winters. This may simply result from a high noise-to-signal ratio in the annual biometric data for such a small species (with therefore little variation to measure) collected by multiple individuals; there is evidence from both cliff swallows *Petrochelidon pyrrhonota* [25] and sand martins *Riparia riparia* [24] that extreme weather events do result in differential mortality of individuals in relation to body size. Our hypothesis that the observed spatial variation in wren body size may underlie the local adaptation of wrens to climate therefore has some support from our analysis, and from other studies. However, given the lack of a significant interaction between the change in mass and region (table 2), more detailed work examining how the impact of severe weather events on the survival of wrens varies with body size is required to confirm this. The fact that variation in the number of frost days at which wren populations reach stability between regions correlates with 1961–1990 regional climate (FD_H_) suggests that at least part of the mechanism underpinning adaptation may operate over a longer timescale than through relatively rapid changes in body size in response to cold winters. This could include variation in behaviour, diet, physiology or other mechanisms [49].

For the spatial gradient in wren body size to exist, there must be a cost to being too large in areas of milder climate that will provide counter-selection in warmer climates (although see [28]). This could result from the greater maintenance costs associated with larger body size [50], larger predation risk due to slower flight speeds and lower agility [51–53] or increased resource requirements of nestlings in order to develop large body size [54,55]. Further work is required to identify this cost.

Our finding that wrens were less common in regions of the UK where the number of winter frost days are higher might seem counterintuitive given the evidence for populations being adapted to local climate. However, such an effect may occur through a number of possible mechanisms. Firstly, spatial variation in density may not be limited by winter climate influencing survival, but other demographic parameters, such as spatial variation in reproductive success, which could be linked to climate through variation in food resources or other ecological parameters. Certainly, there is likely to be latitudinal variation in reproductive outputs, which have not yet been examined in wrens, but do occur in other passerines (e.g. [56]), and could result in an apparent correlation between abundance and winter temperature, even if not causal. Secondly, if there is a lag in the time it takes for a population to adapt to a changing climate, which seems likely, then the observed pattern between abundance and mean winter severity could simply be a consequence of that lag. Thus, a reduction in winter severity would lead to a wren population increase until counter-selection stabilized the population at a higher level. Conversely, any increase in winter severity would reduce the population until locally adapted to the new climate, when again, it would stabilize at a lower level.

Evolution is one of the three mechanisms by which a population can persist in response to climate change (the others being dispersal and phenotypic plasticity) [13]. The extent to which populations may adapt to climate change through evolution will depend upon trait heritability, the strength of selection and the rate of environmental change. Although we have not quantified these parameters, we present evidence that is consistent with winter wren populations being locally adapted to their climate, and partial evidence that this is driven by spatial variation in body size. Relatively few studies have identified genetic responses to climate change [57,58]. Wrens may show local adaptation to climate because there is a strong relationship between climate (winter cold) and fitness coupled with a high potential for local evolution to occur through fast generation times and low dispersal. This suggests that although much research on evolutionary adaptation to climate change has focused on phenological change, studies considering the selection of traits, such as body size, which affect individual fitness in response to climate-driven mortality, such as extreme events, may be equally or more useful. In such instances, work to quantify heritability and the strength of selection associated with these traits will be required to quantify the extent to which evolution may help species adapt to future climate change.

## Supplementary Material

Supplementary material

## Data accessibility

The associated data of this study are openly accessible on the Dryad online data repository at: http://dx.doi.org/10.5061/dryad.d47c1.

## References

[1] Parmesan C 1996 Climate and species' range. Nature 382, 765–766. (doi:10.1038/382765a0)

[2] Gaston KJ 2003 The structure and dynamics of geographic ranges. Oxford, UK: Oxford University Press.

[3] Huntley B, Collingham YC, Willis SG, Green RE 2008 Potential impacts of climatic change on European breeding birds. PLoS ONE 3, e1439 (doi:10.1371/journal.pone.0001439) 18197250 10.1371/journal.pone.0001439 PMC2186378

[4] Root TL, Price JT, Hall KR, Schneider SH, Rosenzweig C, Pounds JA 2003 Fingerprints of global warming on wild animals and plants. Nature 421, 57–60. (doi:10.1038/nature01333) 12511952 10.1038/nature01333

[5] Chen I-C, Hill JK, Ohlemüller R, Roy DB, Thomas CD 2011 Rapid range shifts of species associated with high levels of climate warming. Science 333, 1024–1026. (doi:10.1126/science.1206432) 21852500 10.1126/science.1206432

[6] Sunday JM, Bates AE, Dulvy NK 2012 Thermal tolerance and the global redistribution of animals. Nat. Clim. Change 2, 686–690. (doi:10.1038/nclimate1539)

[7] Garant D, Kruuk LEB, Wilkin TA, McCleery RH, Sheldon BC 2005 Evolution driven by differential dispersal within a wild bird population. Nature 433, 60–65. (doi:10.1038/nature03051) 15635409 10.1038/nature03051

[8] Liker A, Papp Z, Bókony V, Lendvai AZ 2008 Lean birds in the city: body size and condition of house sparrows along the urbanization gradient. J. Anim. Ecol. 77, 789–795. (doi:10.1111/j.1365-2656.2008.01402.x) 18479344 10.1111/j.1365-2656.2008.01402.x

[9] Sheldon BC 2010 Genetic perspectives on the evolutionary consequences of climate change in birds. In Effects of climate change on birds (ed. Møller AP), pp. 149–168. Oxford, UK: Oxford University Press.

[10] Thomas CD et al. 2004 Extinction risk from climate change. Nature 427, 145–148. (doi:10.1038/nature02121) 14712274 10.1038/nature02121

[11] Bellard C, Bertelsmeier C, Leadley P, Thuiller W, Courchamp F 2012 Impacts of climate change on the future of biodiversity. Ecol. Lett. 15, 365–377. (doi:10.1111/j.1461-0248.2011.01736.x) 22257223 10.1111/j.1461-0248.2011.01736.x PMC3880584

[12] Warren R et al. 2013 Quantifying the benefit of early climate change mitigation in avoiding biodiversity loss. Nat. Clim. Change 3, 678–682. (doi:10.1038/nclimate1887)

[13] Chevin L-M, Lande R, Mace GM 2010 Adaptation, plasticity, and extinction in a changing environment: towards a predictive theory. PLoS Biol. 8, e1000357 (doi:10.1371/journal.pbio.1000357) 20463950 10.1371/journal.pbio.1000357 PMC2864732

[14] Visser ME, Both C 2005 Shifts in phenology due to global climate change: the need for a yardstick. Proc. R. Soc. B 272, 2561–2569. (doi:10.1098/rspb.2005.3356) 10.1098/rspb.2005.3356 PMC1559974 16321776

[15] Nussey DH, Wilson AJ, Brommer JE 2007 The evolutionary ecology of individual phenotypic plasticity in wild populations. J. Evol. Biol. 20, 831–844. (doi:10.1111/j.1420-9101.2007.01300.x) 17465894 10.1111/j.1420-9101.2007.01300.x

[16] Charmantier A, McCleery RH, Cole LR, Perrins C, Kruuk LE, Sheldon BC 2008 Adaptive phenotypic plasticity in response to climate change in a wild bird population. Science 320, 800–803. (doi:10.1126/science.1157174) 18467590 10.1126/science.1157174

[17] Husby A, Nussey DH, Visser ME, Wilson AJ, Sheldon BC, Kruuk LEB 2010 Contrasting patterns of phenotypic plasticity in reproductive traits in two great tit (*Parus major*) populations. Evolution 64, 2221–2237. (doi:10.1111/j.1558-5646.2010.00991.x) 20298465 10.1111/j.1558-5646.2010.00991.x

[18] Phillimore AB, Stålhandske S, Smithers RJ, Bernard R 2012 Dissecting the contributions of plasticity and local adaptation to the phenology of a butterfly and its host plants. Am. Nat. 180, 655–670. (doi:10.1086/667893) 23070325 10.1086/667893

[19] Phillimore AB, Hadfield JD, Jones OR, Smithers RJ 2010 Differences in spawning date between populations of common frog reveal local adaptation. Proc. Natl Acad. Sci. USA 107, 8292–8297. (doi:10.1073/pnas.0913792107) 20404185 10.1073/pnas.0913792107 PMC2889515

[20] van Asch M, Salis L, Holleman LJM, van Lith B, Visser ME 2013 Evolutionary response of the egg hatching date of a herbivorous insect under climate change. Nat. Clim. Change 3, 244–248. (doi:10.1038/nclimate1717)

[21] Martin TE 2007 Climate correlates of 20 years of trophic changes in a high-elevation riparian system. Ecology 88, 367–380. (doi:10.1890/0012-9658(2007)88[367:CCOYOT]2.0.CO;2) 17479755 10.1890/0012-9658(2007)88[367:ccoyot]2.0.co;2

[22] Reed TE, Grøtan V, Jenouvrier S, Sæther B-E, Visser ME 2013 Population growth in a wild bird is buffered against phenological mismatch. Science 340, 488–491. (doi:10.1126/science.1232870) 23620055 10.1126/science.1232870

[23] Lande R, Arnold SJ 1983 The measurement of selection on correlated characters. Evolution 37, 1210–1226. (doi:10.2307/2408842) 10.1111/j.1558-5646.1983.tb00236.x 28556011

[24] Jones G 1987 Selection against large size in the sand martin *Riparia riparia* during a dramatic population crash. Ibis 129, 274–280. (doi:10.1111/j.1474-919X.1987.tb03208.x)

[25] Brown CR, Brown MB 1998 Intense natural selection on body size and wing and tail asymmetry in cliff swallows during severe weather. Evolution 52, 1461–1475. (doi:10.2307/2411315) 10.1111/j.1558-5646.1998.tb02027.x 28565386

[26] Grant BR, Grant PR 1993 Evolution of Darwin's finches caused by a rare climatic event. Proc. R. Soc. Lond. B 251, 111–117. (doi:10.1098/rspb.1993.0016)

[27] Clark JA 2009 Selective mortality of waders during severe weather. Bird Study 56, 96–102. (doi:10.1080/00063650802648465)

[28] Brown MB, Brown CR 2011 Intense natural selection on morphology of cliff swallows (*Petrochelidon pyrrhonota*) a decade later: did the population move between adaptive peaks? Auk 128, 69–77. (doi:10.1525/auk.2011.10219)

[29] Greenwood JJD, Baillie SR 1991 Effects of density-dependence and weather on population changes of English passerines using a non-experimental paradigm. Ibis 133, 121–133. (doi:10.1111/j.1474-919X.1991.tb07675.x)

[30] Robinson RA, Baillie SR, Crick HQP 2007 Weather-dependent survival: implications of climate change for passerine population processes. Ibis 149, 357–364. (doi:10.1111/j.1474-919X.2006.00648.x)

[31] Pearce-Higgins JW, Eglington SM, Martay B, Chamberlain DE 2015 Drivers of climate change impacts on bird communities. J. Anim. Ecol. 84, 943–954. (doi:10.1111/1365-2656.12364) 25757576 10.1111/1365-2656.12364

[32] Peach W, Feu CD, McMeeking J 1995 Site tenacity and survival rates of wrens *Troglodytes troglodytes* and treecreepers *Certhia familiaris* in a Nottinghamshire wood. Ibis 137, 497–507. (doi:10.1111/j.1474-919X.1995.tb03259.x)

[33] Paradis E, Baillie SR, Sutherland WJ, Gregory RD 1998 Patterns of natal and breeding dispersal in birds. J. Anim. Ecol. 67, 518–536. (doi:10.1046/j.1365-2656.1998.00215.x)

[34] Newson SE, Evans KL, Noble DG, Greenwood JJD, Gaston KJ 2008 Use of distance sampling to improve estimates of national population sizes for common and widespread breeding birds in the UK. J. Appl. Ecol. 45, 1330–1338. (doi:10.1111/j.1365-2664.2008.01480.x)

[35] Harris SJ et al. 2014 The Breeding Bird Survey 2013. BTO Res. Rep., 658. Thetford, UK: British Trust for Ornithology.

[36] Baillie SR, Wernham CV, Clark JA 1999 Development of the British and Irish ringing scheme and its role in conservation biology. Ringing Migr. 19, 5–19. (doi:10.1080/03078698.1999.9674207)

[37] Gosler AG 2002 Strategy and constraint in the winter fattening response to temperature in the great tit *Parus major*. J. Anim. Ecol. 71, 771–779. (doi:10.1046/j.1365-2656.2002.00642.x)

[38] Bates D et al. 2014 Package ‘lme4’. Vienna, Austria: R Foundation for Statistical Computing.

[39] R Core Development Team. 2014 R: A language and environment for statistical computing. Vienna, Austria: R Foundation for Statistical Computing.

[40] Freckleton RP, Watkinson AR, Green RE, Sutherland WJ 2006 Census error and the detection of density dependence. J. Anim. Ecol. 75, 837–851. (doi:10.1111/j.1365-2656.2006.01121.x) 17009748 10.1111/j.1365-2656.2006.01121.x

[41] Burnham KP, Anderson DR 2002 Model selection and multimodel inference: a practical information-theoretic approach. New York, NY: Springer Science & Business Media.

[42] Renwick AR, Johnston A, Joys A, Newson SE, Noble DG, Pearce-Higgins JW 2012 Composite bird indicators robust to variation in species selection and habitat specificity. Ecol. Indicators 18, 200–207. (doi:10.1016/j.ecolind.2011.11.008)

[43] Williamson K 1969 Habitat preferences of the wren on English farmland. Bird Study 16, 53–59. (doi:10.1080/00063656909476216)

[44] Karell P, Ahola K, Karstinen T, Valkama J, Brommer JE 2011 Climate change drives microevolution in a wild bird. Nat. Commun. 2, 208 (doi:10.1038/ncomms1213) 21343926 10.1038/ncomms1213 PMC3105316

[45] Bennie J, Kubin E, Wiltshire A, Huntley B, Baxter R 2010 Predicting spatial and temporal patterns of bud-burst and spring frost risk in north-west Europe: the implications of local adaptation to climate. Glob. Change Biol. 16, 1503–1514. (doi:10.1111/j.1365-2486.2009.02095.x)

[46] Hadfield JD, Wilson AJ, Garant D, Sheldon BC, Kruuk LEB 2010 The misuse of BLUP in ecology and evolution. Am. Nat. 175, 116–125. (doi:10.1086/648604) 19922262 10.1086/648604

[47] Johnston RF, Selander RK 1973 Evolution in the house sparrow. III. Variation in size and sexual dimorphism in Europe and North and South America. Am. Nat. 107, 373–390. (doi:10.1086/282841)

[48] Cawthorne RA, Marchant JH 1980 The effects of the 1978/79 winter on British bird populations. Bird Study 27, 163–172. (doi:10.1080/00063658009476675)

[49] Piersma T, van Gils JA 2011 The flexible phenotype. A body-centred integration of ecology, physiology, and behaviour. Oxford, UK: Oxford University Press.

[50] Witter MS, Cuthill IC 1993 The ecological costs of avian fat storage. Phil. Trans. R. Soc. Lond. B 340, 73–92. (doi:10.1098/rstb.1993.0050) 8099746 10.1098/rstb.1993.0050

[51] Andersson M, Norberg RA 1981 Evolution of reversed sexual size dimorphism and role partitioning among predatory birds, with a size scaling of flight performance. Biol. J. Linn. Soc. 15, 105–130. (doi:10.1111/j.1095-8312.1981.tb00752.x)

[52] Alerstam T, Lindström Å 1990 Optimal bird migration: the relative importance of time, energy, and safety. In Bird Migr. SE - 22 (ed. Gwinner E), pp. 331–351. Berlin, Germany: Springer.

[53] Gosler AG, Greenwood JJD, Perrins C 1995 Predation risk and the cost of being fat. Nature 377, 621–623. (doi:10.1038/377621a0)

[54] Price TD, Grant PR 1984 Life history traits and natural selection for small body size in a population of Darwin's finches. Evolution 38, 483–494. (doi:10.2307/2408698) 10.1111/j.1558-5646.1984.tb00314.x 28555964

[55] Weathers WW 1992 Scaling nestling energy requirements. Ibis 134, 142–153. (doi:10.1111/j.1474-919X.1992.tb08391.x)

[56] Sanz JJ 1998 Effects of geographic location and habitat on breeding parameters of great tits. Auk 115, 1034–1051. (doi:10.2307/4089520)

[57] Gienapp P, Teplitsky C, Alho JS, Mills JA, Merilä J 2008 Climate change and evolution: disentangling environmental and genetic responses. Mol. Ecol. 17, 167–178. (doi:10.1111/j.1365-294X.2007.03413.x) 18173499 10.1111/j.1365-294X.2007.03413.x

[58] Merilä J 2012 Evolution in response to climate change: in pursuit of the missing evidence. BioEssays 34, 811–818. (doi:10.1002/bies.201200054) 22782862 10.1002/bies.201200054

