# Supplementary material for: Winter wren populations show adaptation to local climate
Source: R Soc Open Sci. 2016 Jun 29;3(6):160250. doi: 10.1098/rsos.160250 (PMC4929917; doi:10.1098/rsos.160250)
Supplement: Supplementary material [file rsos160250supp1.docx]

**Supplementary material for: Winter wren populations show adaptation to local climate.**

**
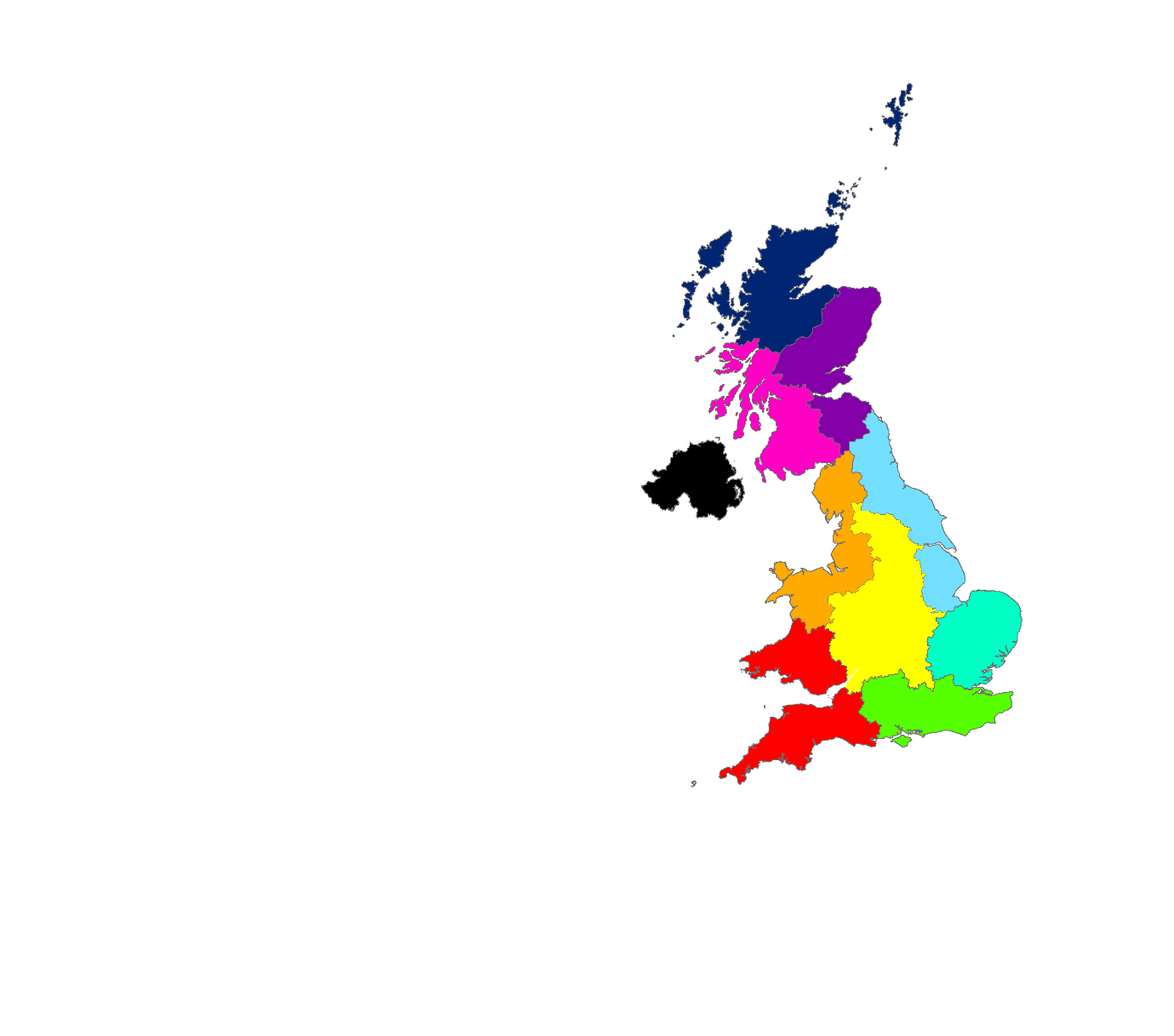
**

**Figure S1:** Boundaries of the 10 Met Office climate regions.

**
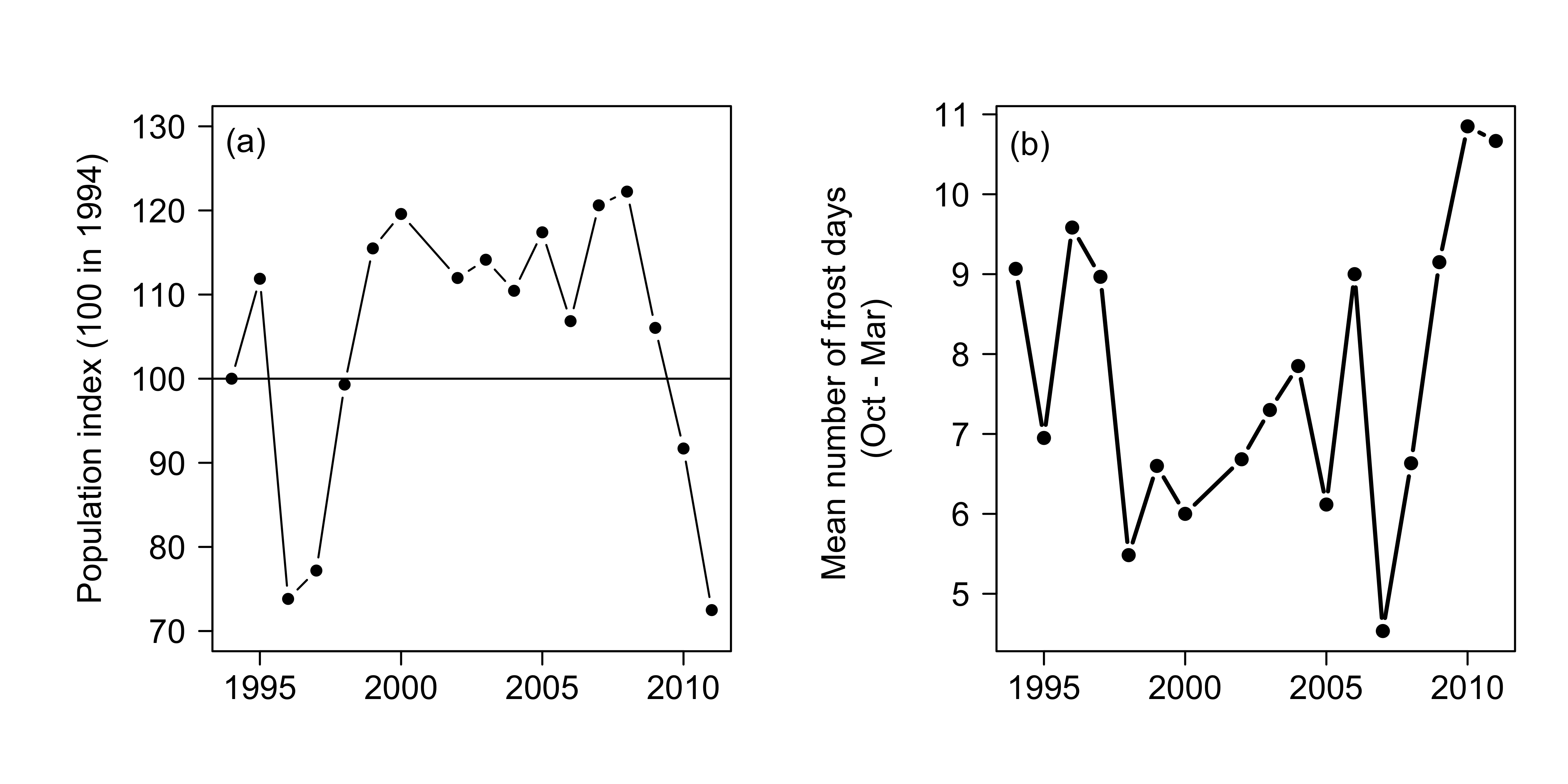
Figure S2:** Annual variation in UK a) wren BBS population index (Harris *et al.* 2015) and b) mean number of winter frost days (Oct – Mar) from 1994 to 2011.

**
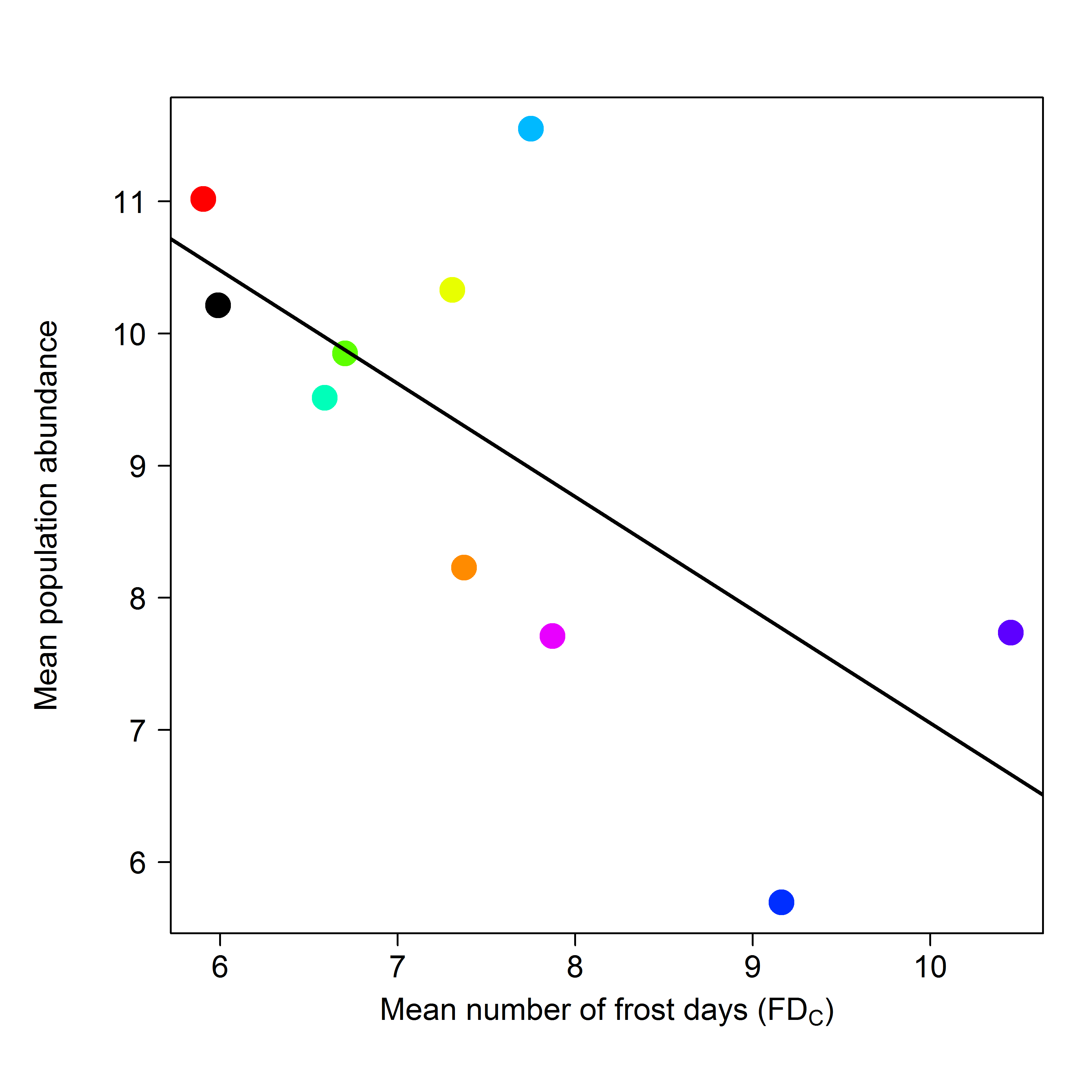
**

**Figure S3:** Relationship between population abundance and regional mean number of winter frost days (F_c_) on woodland BBS sites (those with > 50% of woodland transect sections) (Pearson correlation: r = -0.67, n = 10, p = 0.03). Each data point corresponds to a regional mean, with the number of winter frost days (FD_C_) calculated as the overall mean of the winter frost day index (FD) from 1994 to 2011, while the mean population abundance from 1994 to 2011 (number of wrens per BBS site) was predicted from the results of a GLMM (see materials and methods).


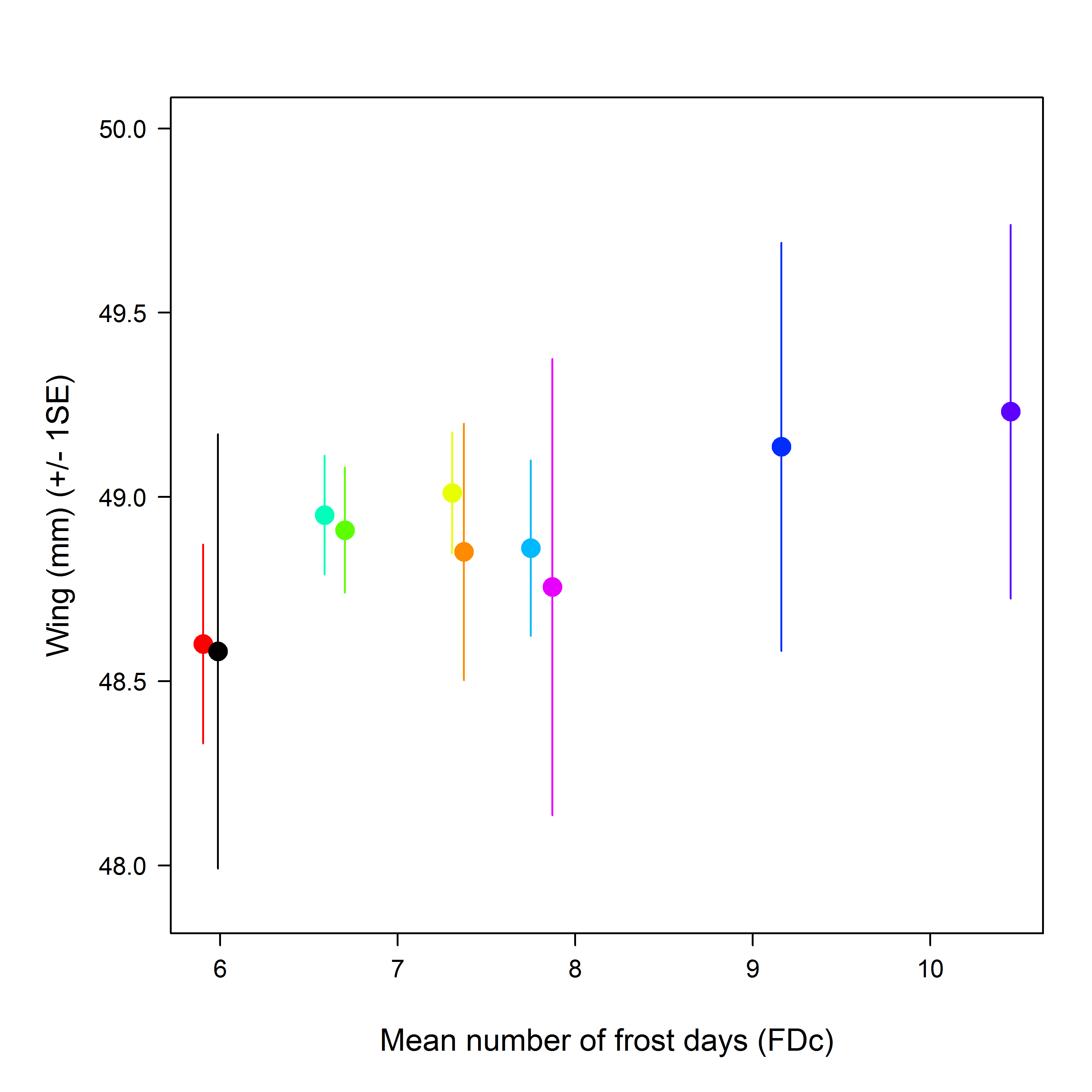


**Figure S4:** Relationship between regional mean wing length and the mean number of winter frost days (F_c_) (Pearson correlation: r = 0.82, n = 9, p = 0.003). Each data point corresponds to a regional mean, with wing length calculated as a mean of the annual means and the number of winter frost days (FD_C_) calculated as the overall mean of the winter frost day index (FD) from 1994 to 2011.

**Table S1:** Results of a GLMM of variation in wren population growth rate in relation to the winter frost day index (FD) (average number of frost days per month (Oct – Mar) in 1994-2011) and the historic regional climate (1961 – 1990) (FD_H_) (an overall mean of the winter frost day index from 1961 to 2011). The interaction between FD and FD_H_ demonstrates that population sensitivity to winter weather varies significantly and predictable with winter climate.

| Parameter | Estimate (SE) | z-value | p-value |
| --- | --- | --- | --- |
| FD_H_ | 0.03 (0.02) | 1.72 | 0.08 |
| FD | -0.03 (0.01) | -2.84 | 0.005 |
| Count in previous year | -0.04 (0.001) | -71.75 | <0.001 |
| FD_H_* FD | -0.003 (0.001) | -2.66 | 0.007 |

**Table S2:** Results of GLMs of regional variation in body mass and wing length of wren populations.

| Variable | Parameter | Sum sq | df | F-value | p-value |
| --- | --- | --- | --- | --- | --- |
| Body mass | Region | 224.6 | 9 | 28.9 | <0.001 |
|  | Residuals | 26445.9 | 30700 |  |  |
| Wing length | Region | 185.0 | 9 | 4.9 | <0.001 |
|  | Residuals | 151252 | 36332 |  |  |

**Table S3:** Results of a GLM of variation in annual change in the mean regional wing length of wren populations.

| Parameter | Χ^2^ | df | p-value |
| --- | --- | --- | --- |
| Region | 2.25 | 8 | 0.97 |
| Abundance | 0.37 | 1 | 0.54 |
| FD | 0.41 | 1 | 0.52 |
| Region* FD | 7.33 | 8 | 0.50 |

**References**

Harris, S.J., Massimino, D., Newson, S.E., Eaton, M.A., Balmer, D.E., Noble, D.G., Musgrove, A.J., Gillings, S., Procter, D. & Pearce-Higgins, J.W. (2015). The Breeding Bird Survey 2014. *BTO research report*, **673**.

**Data:**

**Table 1:** Regional annual population growth rate.

| **Region** | **94-95** | **95-96** | **96-97** | **97-98** | **98-99** | **99-00** | **00-01** | **01-02** | **02-03** | **03-04** | **04-05** | **05-06** | **06-07** | **07-08** | **08-09** | **09-10** | **10-11** |
| --- | --- | --- | --- | --- | --- | --- | --- | --- | --- | --- | --- | --- | --- | --- | --- | --- | --- |
| South-west | 1.19 | 0.83 | 0.96 | 1.17 | 1.20 | 1.14 | 0.97 | 1.32 | 1.05 | 1.03 | 1.13 | 0.91 | 1.15 | 1.08 | 0.94 | 0.97 | 0.87 |
| North-west | 1.20 | 0.77 | 0.95 | 1.18 | 1.17 | 1.10 | 1.00 | 1.18 | 1.10 | 1.10 | 1.14 | 1.06 | 1.17 | 1.08 | 0.97 | 0.98 | 0.89 |
| Midlands | 1.13 | 0.76 | 1.02 | 1.16 | 1.21 | 1.09 | 0.96 | 1.11 | 1.08 | 1.00 | 1.12 | 1.02 | 1.12 | 1.08 | 0.92 | 1.00 | 0.85 |
| South-east | 1.24 | 0.88 | 0.87 | 1.11 | 1.25 | 1.09 | 1.06 | 1.13 | 1.19 | 1.07 | 1.16 | 0.96 | 1.13 | 1.08 | 1.04 | 1.03 | 1.02 |
| East | 1.09 | 0.77 | 0.85 | 1.18 | 1.27 | 1.11 | 0.97 | 1.12 | 1.06 | 0.95 | 1.16 | 0.98 | 1.19 | 1.07 | 0.99 | 0.97 | 1.03 |
| North-east | 1.19 | 0.78 | 1.06 | 1.16 | 1.18 | 1.16 | 0.92 | 1.22 | 1.16 | 1.01 | 1.11 | 1.07 | 1.24 | 1.16 | 0.89 | 1.02 | 0.88 |
| North Scotland | 1.16 | 0.70 | 1.55 | 1.25 | 1.08 | 1.17 | 0.96 | 1.05 | 1.04 | 1.26 | 1.20 | 1.07 | 1.29 | 1.14 | 0.91 | 0.63 | 0.85 |
| East Scotland | 1.25 | 0.77 | 1.15 | 1.25 | 1.09 | 1.23 | 0.83 | 1.22 | 1.23 | 1.24 | 1.17 | 1.09 | 1.19 | 1.28 | 0.86 | 0.75 | 0.83 |
| West Scotland | 1.34 | 0.56 | 0.93 | 1.35 | 1.43 | 1.24 | 0.73 | 1.10 | 1.22 | 1.31 | 1.45 | 1.16 | 1.23 | 1.28 | 1.13 | 0.82 | 0.84 |
| Northern Ireland | 0.98 | 0.91 | 1.03 | 1.36 | 1.06 | 1.33 | NA | NA | 1.13 | 1.07 | 1.18 | 1.12 | 1.14 | 1.02 | 1.08 | 0.90 | 0.57 |

**Table 2:** Regional mean number of frost days when populations are stable.

| **Region** | **Number of frost days when population is stable** |
| --- | --- |
| South-west | 7.01 |
| North-west | 8.85 |
| Midlands | 8.86 |
| South-east | 8.02 |
| East | 7.89 |
| North-east | 9.40 |
| North Scotland | 10.74 |
| East Scotland | 12.12 |
| West Scotland | 10.37 |
| Northern Ireland | 7.60 |

**Table 3:** Regional mean wren relative abundance.

| **Region** | **Mean wren population abundance (1994 - 2011)** |
| --- | --- |
| South-west | 8.68 |
| North-west | 6.47 |
| Midlands | 7.02 |
| South-east | 7.50 |
| East | 6.20 |
| North-east | 5.43 |
| North Scotland | 4.04 |
| East Scotland | 5.30 |
| West Scotland | 5.89 |
| Northern Ireland | 10.11 |

**Table 4:** Regional mean wren body mass (g).

| **Region** | **Mean wren body mass (1994 – 2011)** |
| --- | --- |
| South-west | 10.08 |
| North-west | 10.27 |
| Midlands | 10.18 |
| South-east | 10.06 |
| East | 10.10 |
| North-east | 10.27 |
| North Scotland | 10.80 |
| East Scotland | 10.54 |
| West Scotland | 10.56 |
| Northern Ireland | 10.44 |
